# Breast cancer risk in elderly women with systemic autoimmune rheumatic diseases: a population-based case–control study

**DOI:** 10.1038/sj.bjc.6604906

**Published:** 2009-02-03

**Authors:** S M Gadalla, S Amr, P Langenberg, M Baumgarten, W F Davidson, C Schairer, E A Engels, R M Pfeiffer, J J Goedert

**Affiliations:** 1 Division of Cancer Epidemiology and Genetics, National Cancer Institute, Bethesda, MD 20892, USA; 2 Department of Epidemiology and Preventive Medicine, School of Medicine, University of Maryland, Baltimore, MD 21201, USA; 3 Department of Microbiology and Immunology, School of Medicine, University of Maryland, Baltimore, MD 21201, USA

**Keywords:** rheumatic diseases, autoimmune diseases, breast cancer

## Abstract

Systemic autoimmune rheumatic diseases (SARDs) are chronic inflammatory and immuno-modulatory conditions that have been suggested to affect cancer risk. Using the Surveillance, Epidemiology and End Results–Medicare-linked database, women aged 67–99 years and diagnosed with incident breast cancer in 1993–2002 (*n*=84 778) were compared with an equal number of age-matched cancer-free female controls. Diagnoses of SARDs, including rheumatoid arthritis (RA, *n*=5238), systemic lupus erythematosus (SLE, *n*=340), Sjogren's syndrome (*n*=374), systemic sclerosis (*n*=128), and dermatomyositis (*n*=31), were determined from claim files for individuals from age 65 years to 1 year before selection. Associations of SARD diagnoses with breast cancer, overall and by oestrogen receptor (ER) expression, were assessed using odds ratio (OR) estimates from multivariable logistic regression models. The women diagnosed with RA were less likely to develop breast cancer (OR=0.87, 95% confidence interval (CI)=0.82–0.93). The risk reduction did not differ by tumour ER-status (OR=0.83, 95% CI=0.78–0.89 for ER-positive *vs* OR=0.91, 95% CI=0.81–1.04 for ER-negative, *P* for heterogeneity=0.14). The breast cancer risk was not associated with any of the other SARDs, except for a risk reduction of ER-negative cases (OR=0.49, 95% CI=0.26–0.93) among women with SLE. These findings suggest that systemic inflammation may affect breast epithelial neoplasia.

Breast cancer (BC), the commonest malignancy and the second leading cause of cancer death among American women (American Cancer Society, 2007), is heterogeneous, particularly with respect to expressions of oestrogen and progesterone receptors. Oestrogen receptor (ER) status is of special interest because of its clinical implication (Andry *et al*, 1989; Hess *et al*, 2003). The fact that known BC risk factors account for only 45–55% of the cases (Willett *et al*, 2004) indicates the need for further research. Systemic autoimmune rheumatic diseases (SARDs) predominantly affect women (Cooper and Stroehla, 2003). They include rheumatoid arthritis (RA), systemic lupus erythematosus (SLE), Sjogren's syndrome, systemic sclerosis, and dermatomyositis (Marrow *et al*, 1999). Typically, these are associated with activation of auto-reactive T and B lymphocytes and release of pro-inflammatory cytokines, which may alter cancer risk (Salazar-Onfray *et al*, 2007).

Several studies of SARDs and breast cancer risk have had inconsistent results (Hill *et al*, 2003; Chatterjee *et al*, 2005; Derk *et al*, 2006; Kontos and Fentiman, 2008; Smitten *et al*, 2008), which may reflect limitations such as small sample size, short time of follow-up, selection bias in hospital-based cohorts, detection bias, or reverse causality with a short interval between SARD diagnoses and BC. In addition, if any BC risk associated with SARDs is ER-specific, the distribution of ER status in the studied populations might lead to conflicting results.

We conducted a population-based case–control study using the Surveillance, Epidemiology and End Results (SEER)–Medicare-linked database to evaluate whether a history of SARD diagnoses was associated with BC risk overall and by ER status of the tumour.

## Subjects and methods

The SEER–Medicare data linkage, a collaboration of the National Cancer Institute and the Center for Medicare and Medicaid Services, was created by linking two large population-based data sources: the SEER cancer registry system and the Medicare enrolment and claim files. Detailed description of the database is available elsewhere (Warren *et al*, 2002). Briefly, it includes all incident cancer cases recorded by SEER registries, currently encompassing ∼25% of the US population, plus a 5% random sample of all Medicare beneficiaries residing in SEER areas to serve as population-based controls. The Medicare part of the linkage includes data from inpatient claims since 1986 and from all other types of claims (outpatient, physician, home health, and hospice services) since 1991. The database does not include claims for Medicare beneficiaries during enrolment in a health maintenance organisation (HMO). Clinical diagnoses are coded using the International Classification of Diseases, 9th Revision (ICD-9) codes (US Public Health Service, 1996).

From the SEER database, we included all women with incident primary invasive adenocarcinoma of the breast (ICD-O-3 C500-C509; histology codes 8140, 8201, 8211, 8480, 8500, 8501, 8503, 8504, 8520, 8521, 8522, 8523, 8524, 8530, 8541, and 8543; and behaviour code 3) who were diagnosed in 1993–2002, with no previous cancer of any type; BC cases diagnosed only at autopsy or by death certificate were excluded.

Female controls who were alive and cancer free as of 1 July of the calendar year of case selection were selected at random, with replacement, from the 5% sample of the Medicare beneficiaries who resided in SEER areas. To ensure availability of claims data, cases and controls had to be 67–99 years of age, selected in 1993 or later, and have at least 12 months of simultaneous part A and B coverage (and no HMO coverage) before the selection date. Controls were frequency matched in 1 : 1 ratio to cases according to the calendar year of diagnosis and age in three categories (67–74, 75–84, and 85+). Women who became BC cases could be selected as controls until they were diagnosed with the cancer.

Participants were considered to have SARDs if they had at least one inpatient or two outpatient/physician claims (with a minimum interval of 30 days between claims) for any of the following diagnoses: RA (ICD-9 714.0, 714.1, 714.2, 714.3, 714.81, or V82.1), SLE (710.0), systemic sclerosis (710.1), Sjogren's syndrome (710.2), or dermatomyositis (710.3). Women who met the definition for more than one condition were included in a separate category, multiple SARDs. History of SARDs was ascertained from Medicare claim files, up to 12 months before case–control selection date.

Variables assessed as potential confounders of associations between BC and SARDs included age, race, socio-economic status, region of residence, history of mammography, number of physician visits 12–24 months before selection, and earlier use of immunosuppressive medications. We used the 1990 census median annual household income in the study participants’ zip code of residence as a proxy measure of individuals’ socioeconomic status. The regions of residence, based on the location of the SEER registry, were categorised as western, northeastern, north-central, and southern (Table 1). History of mammography was defined as any mammography claim recorded from 12 months to a maximum of 48 months before case–control selection. History of immunosuppressive therapy was defined as any Medicare claim for the following between age 65 years and 12 months before the case–control selection: cyclophosphamide, methotrexate, chlorambucil, azathioprine, cyclosporine, mycophenolate mofetil, sirolimus, tacrolimus, prednisone, prednisolone, methyl prednisolone, and immunosuppressive medication not otherwise specified.

### Statistical analysis

We used unconditional logistic regression to calculate odds ratios (ORs) and 95% confidence intervals (CIs) for the association between BC risk and SARDs (overall and for each condition). We computed the variance of the OR estimates using a robust variance estimator (Zeger and Liang, 1986) to adjust for the correlations between observations when the same participant was selected in different calendar years.

In evaluating BC risk by ER status, we excluded cases with unknown ER expression. We used polytomous logistic regression to estimate ORs and 95% CIs with a robust variance estimator comparing ER-positive cases and ER-negative cases with the cancer-free controls (Anderson *et al*, 2008). A Wald test with one degree of freedom was used to test for the heterogeneity in the estimated regression coefficient for specific SARDs between ER-positive and ER-negative cases.

All final models were adjusted for age and year of selection (matching variables), race, region of residence, median-household income by zip code of residence, history of mammography, and history of immunosuppressive therapy as defined earlier. We also stratified all the models by year of selection in three groups (1993–95, 1996–99, and 2000–02) to investigate whether the estimated associations were affected by secular trends in coding accuracy, mammography screening, or standard of care. In the final models that evaluated overall BC risk associated with SARDs (overall and by condition), we added two interaction terms, age (⩾75 *vs* <75) and race (whites *vs* others), to test whether these associations are modified by age or race.

## Results

The study included 84 778 BC cases and an equal number of cancer-free controls. Most of the cases (78.2%) had known ER status, of whom 84.9% were ER-positive. As shown in Table 1, cases and controls had similar age distribution (median=67 years), region of residence, duration of Medicare coverage (median=101 *vs* 102 months, respectively), and number of visits to physicians from 12 to 24 months before case–control selection (median=12 *vs* 11 visits, respectively). However, more cases than controls were white (88.4 *vs* 83.7%), and had history of mammography 12–48 months before selection (50.5 *vs* 45.4%). Patterns were similar for ER-positive and ER-negative cases, with the exception of race, for which more ER-negative cases than controls were black (9.4 *vs* 7.7%) (Table 1).

Breast cancer cases with unknown ER status were similar to those with known status with respect to SARD distribution, but they were more likely to be diagnosed between 1993 and 1995 (26.5 *vs* 21.4%), have tumours of unknown grade (25.3 *vs* 12.3%), have non-specific morphology (10.8 *vs* 1.5%), and no recorded stage (5.6 *vs* 0.7%). Although statistically significant, absolute differences between cases with known and unknown ER status with respect to age, race, and income were small (<5%) (Supplementary Table S1).

Of all participants, 4.0% (*n*=3033 in BC cases and 3396 in the controls) met the study definition of at least one SARD condition. Among them, 95.2% had only one condition. Of those who had been diagnosed with more than one condition, 84.6% had RA.

In unadjusted analyses, history of having any SARD diagnosis was inversely associated with breast cancer risk (OR=0.89, 95% CI=0.84–0.93). When comparing individual SARD cases with no SARD cases, only RA was significantly associated with a decreased BC risk (OR=0.87, 95% CI=0.82–0.92) (data not shown). Multivariable regression models showed similar results (Table 2). Similar results were also obtained when we stratified the models by year of selection (data not shown). No significant interactions were found with age or race in the association between BC and SARD (overall or for individual condition, data not presented).

When comparing ER-positive and ER-negative cases with cancer-free controls in unadjusted analysis, RA was inversely associated with both ER-positive and ER-negative BC risk (OR=0.83, 95% CI=0.78–0.89, and OR=0.90, 95% CI=0.80–1.03, respectively), whereas SLE was inversely associated only with ER-negative tumours (OR=0.50, 95% CI=0.27–0.95) (data not shown). Multivariable analysis showed similar results (Table 2). Heterogeneity in the estimated ORs of ER-positive and ER-negative BC associated with SARDs was only significant in the case of SLE (OR=1.08, 95% CI=0.85–1.40, and OR=0.49, 95% CI=0.26–0.94, respectively, *P*=0.02). Results obtained from stratified models by year of case–control selection and unstratified models were similar, except for an attenuation of the risk deficit for ER-positive BC with RA in recent years (OR=0.76, 0.83, and 0.85 for 1993–1995, 1996–1999, and 2000–2002, respectively, *P* for trend <0.0001) (Supplementary Table S2).

## Discussion

Our study found a reduced BC risk associated with RA that did not differ by the ER-status of the tumour. We also observed a reduced risk in women with SLE that was confined to ER-negative tumours. None of the other SARDs investigated was associated with BC risk overall or by ER status.

Our observed association for RA agrees with the results of a recent meta-analysis that reported a pooled standardised incidence ratio of 0.84 (95% CI=0.79–0.90) for breast cancer among women with RA (Smitten *et al*, 2008). For SLE, our results are consistent with the overall null associations reported by studies that recruited a wide spectrum of SLE patients (Cibere *et al*, 2001; Ragnarsson *et al*, 2003), but they contrast with the protective effect observed in studies that recruited the SLE patients from inpatient records or tertiary care units, which are likely to over-represent severe SLE cases (Bjornadal *et al*, 2002; Bernatsky *et al*, 2005). Perhaps disease activity, severity, or treatment affects its relationship to BC risk. No earlier study evaluated this association by the tumour ER-specific subtypes.

The variation in BC risk with different SARD conditions might reflect differences in cytokine profiles. For example, high circulating levels of tumour necrosis factor-alpha (TNF-*α*) and transforming growth factor-beta (TGF-*β*) have been reported in patients with RA (Arend and Gabay, 2004), but not in patients with Sjogren's syndrome (Garcic-Carrasco *et al*, 2001; Eriksson *et al*, 2004). Both TNF-*α* and TGF-*β* have been reported to suppress breast cancer cell proliferation, predominantly against ER-positive cells (Sgagias *et al*, 1991; Grimm and Rosen, 2006). On the other hand, SLE patients (Lub-de Hooge *et al*, 2005; Rus *et al*, 2005), and to a lesser extent RA patients (Xie *et al*, 2007), have elevated serum levels of TNF-related apoptosis-inducing ligand (TRAIL). TRAIL has been shown to induce apoptosis in ER-negative, but not in ER-positive cell lines (Rahman *et al*, 2009), perhaps explaining our observed reduced risk of ER-negative, but not of ER-positive, breast cancer in women with SLE. Although plausible, we were not able to rule out the possibility that the observed inverse association with RA might be confounded by the use of nonsteroidal anti-inflammatory drugs (NSAIDs). The NSAIDs seem to have a protective effect against BC (Coogan *et al*, 1999; Johnson *et al*, 2002; Harris *et al*, 2003; Takkouche *et al*, 2008). However, the generally null associations with SARD conditions, with the notable exception of RA, suggest that use of NSAIDs does not explain the observed associations.

Possible differences in known BC risk factors that were not available in our study, such as parity, age at first birth, obesity, alcohol consumption, and use of hormone therapy, should also be considered. It seems unlikely that they completely explain the observed associations, as these are the risk factors for ER-positive, but not for ER-negative tumours (Chen and Colditz, 2007). In addition, the low body mass index (BMI) prevalent in women with RA is mainly because of muscle wasting rather than low body fat (Munro and Capell, 1997), and thus is less likely to affect levels of endogenous oestrogens, which are thought to mediate the association between BMI and BC. Also, these factors did not completely confound the association between SLE and BC in an earlier study (Bernatsky *et al*, 2003).

Our study strengths include population-based sampling of the participants and a large sample size. The SEER–Medicare-linked database allowed the identification of incident breast cancer cases and provided detailed tumour information. In addition, we captured SARD conditions from inpatient, outpatient, and physician claims, minimising selection bias by capturing the whole spectrum of the diseases, including the one-quarter of SARD patients who were treated only as outpatients. Exclusion of the 1 year before case–control selection minimised the possibility of reverse causality and detection bias that could result from increased medical surveillance around the time of cancer diagnosis.

Study limitations include the lack of information on important BC risk factors such as obesity and parity, as well as detailed medical information about the SARD conditions, including severity, duration, and detailed treatment history. We adjusted for the use of immunosuppressive medications; however, underestimation of the actual use is expected, especially for the less expensive oral medications, such as corticosteroids, because of the medication coverage rules of Medicare. The missing information on ER expression for one-fifth of the BC cases might be a source of bias. However, the equal distribution of SARD conditions in BC cases with known and unknown ER status suggests that substantial differential bias is unlikely.

In conclusion, this study provides evidence for a reduced risk of BC in older women with RA and also suggests that older women with SLE may be at reduced risk for ER-negative BC, suggesting a possible role for systemic mediators of inflammation and immunity against BC cells.

## Figures and Tables

**Table 1:** Characteristics of breast cancer cases (overall and by ER status) and controls

	**Controls (*N*=84 778)**	**All cases (*N*=84 778)**	**ER-positive cases^a^** **(*N*=56 296)**	**ER-negative cases^a^** **(*N*=9991)**
	**%**			
*Age in years (median)*	(76.0)	(76.0)	(76.0)	(75.0)
67–70	19.2	19.1	19.2	21.3
71–75	28.0	28.3	28.6	29.4
76–80	24.9	25.3	25.8	24.4
81–84	14.6	13.9	16.4	15.2
85+	13.3	13.3	10.1	9.7
				
*Race*				
White	83.7	88.5	89.8	84.7
Black	7.7	6.4	5.2	9.4
Hispanic	2.4	1.3	1.2	1.5
Others/unknown	6.2	3.8	3.8	4.4
				
*Region of residence* b				
Western	51.2	51.3	52.8	47.9
Northeastern	17.0	17.6	16.6	18.8
North-central	20.1	20.1	20.0	21.1
Southern	11.7	11.0	10.6	12.2
				
*Income* c				
<$25 000	20.8	18.7	18.3	20.2
$25 000–$34 999	28.1	28.1	28.5	28.2
$35 000–$49 999	32.4	33.0	33.0	32.4
⩾$50 000	13.2	14.0	14.2	13.4
Unknown	5.4	6.2	6.0	5.8
				
*Selection year*				
1993–1995	22.5	22.5	21.1	23.3
1996–1999	31.5	31.5	31.9	32.5
2000–2002	46.0	46.0	47.0	44.2
				
*Mammography* d				
Yes	45.4	50.5	52.0	48.7
No	54.6	49.5	48.0	51.3
				
*Months of coverage*^e^ *(median)*	(102.0)	(101.0)	(102.0)	(98.0)
12–23	3.2	3.2	3.3	3.1
24–59	19.2	19.2	19.3	21.1
60+	77.6	77.6	77.4	75.8
				
*Number of physician visits*^f^ *(median)*	(11.0)	(12.0)	(12.0)	(12.0)
0	16.0	15.3	15.2	15.2
1–11	36.7	35.2	35.3	36.8
12–23	23.2	24.4	24.7	23.4
24+	24.1	25.1	24.8	24.6

a ER status is missing for 18 491 breast cancer cases

b Western region includes Hawaii, New Mexico, San Francisco, Seattle, Utah, San Jose, Los Angeles, and Greater California; North eastern region includes Connecticut and New Jersey; North-central includes Detroit and Iowa; Southern region includes Atlanta, rural Georgia, Kentucky, and Louisiana.

c Median household income by residential zip code.

d Ever had mammography 12–48 months before case–control selection.

e Number of months of total simultaneous coverage part A, B, and non-HMO.

f Number of visits to a physician 12–24 months before case–control selection.

**Table 2:** Adjusted association between breast cancer (overall and by ER status) and SARD conditions

	**All cases–control (*N*=84 778)**		**ER-positive cases–control (*N*=56 296)**		**ER-negative cases–control (*N*=9991)**	
**SARD**	***N***	**OR (95% CI)^a^**	***N***	**OR (95% CI)^a^**	***N***	**OR (95% CI)^a^**
None	81 745	Reference	54 343	Reference	9627	Reference
Any	3033	0.88 (0.84–0.93)^b^	1953	0.85 (0.80–0.90)^b^	364	0.91 (0.81–1.02)
						
*By condition*						
Rheumatoid arthritis	2444	0.87 (0.82–0.92)^b^	1554	0.83 (0.78–0.89)^b^	301	0.91 (0.81–1.04)
Systemic lupus	172	0.99 (0.78–1.24)	128	1.08 (0.85–1.40)	10	0.49 (0.26–0.94)^b^
Sjogren's syndrome	196	1.06 (0.85–1.32)	130	1.04 (0.82–1.33)	21	1.01 (0.64–1.61)
Systemic sclerosis	64	0.96 (0.65–1.44)	42	0.93 (0.60–1.43)	7	0.88 (0.39–1.98)
Dermatomyositis	14	0.76 (0.35–1.65)	10	0.80 (0.35–1.85)	<5	0.99 (0.22–4.36)
Multiple SARDs	143	0.78 (0.61–0.99)^b^	89	0.71 (0.54–0.94)^b^	23	1.09 (0.70–1.71)

a Odds ratios (ORs) and 95% confidence intervals (CIs) are adjusted for age, year of selection, race, mammography, region of residence, income, and immunosuppressive therapy.

b *P*<0.05.
