# Supplementary material for: Breast cancer risk in elderly women with systemic autoimmune rheumatic diseases: a population-based case–control study
Source: Br J Cancer. 2009 Feb 3;100(5):817–21. doi: 10.1038/sj.bjc.6604906 (PMC2651404; doi:10.1038/sj.bjc.6604906)
Supplement: Supplementary Tables S.1 and S.2 [file 6604906x1.doc]

Table S.1: Comparison between breast cancer cases with known and unknown ER expression

|  | Unknown ER status  N=18,491  % | Known ER status  N=66,287  % | p-value |
| --- | --- | --- | --- |
| Age (Median)  67-74  75-84  85+  Race  White  Black  Hispanic  Others/Unknown  Income^*^  <$25,000  $25,000-34,999  $35,000-49,999  $>=50,000  Unknown  Selection year  1993-1995  1996-1999  2000-2002  Tumor stage  Localized  Regional  Distant  Unstaged  Tumor grade  Well differentiated  Moderately differentiated  Poorly differentiated  Undifferentiated  Unknown  Tumor morphology  Ductal  Lobular  Mixed  Inflammatory  Adenocarcinoma, NOS  Others^†^  Having SARDs  None  RA  SLE  Sjogren’s syndrome  Systemic sclerosis  Dermatomyositis  Multiple SARDs | (76.0)  39.1  44.7  16.2  86.6  8.4  1.5  3.5  19.1  27.0  33.1  14.0  6.8  26.5  29.7  43.8  64.3  22.0  8.1  5.6  16.5  34.2  22.3  1.7  25.3  64.1  9.6  6.6  1.1  10.8  7.8  96.1  3.2  0.2  0.2  0.1  0.0  0.2 | (76.0)  42.3  45.2  12.5  89.0  5.8  1.2  4.0  18.6  28.4  32.9  14.1  6.0  21.4  32.0  46.6  69.1  25.6  4.6  0.7  19.8  40.3  26.0  1.6  12.3  72.5  10.7  8.8  0.7  1.5  5.9  96.5  2.8  0.2  0.2  0.1  0.0  0.2 | <0.0001  <0.0001  <0.0001  <0.0001  <0.0001  <0.0001  <0.0001  0.18 |

^*^ Median household income by Zip code of residency

^†^Tubular, mucinous or adenocarcinoma with Paget disease

Table S.2: Adjusted association between breast cancer by estrogen receptor (ER) status and SARD conditions, compared to

cancer- free controls stratified by selection year

| **SARD** | ER-positive/control  N=56,296 | | | ER-negative/control  N=9,991 | | |
| --- | --- | --- | --- | --- | --- | --- |
|  | 1993-1995  N=11,883 | 1996-1999  N=17,942 | 2000-2002  N=26,471 | 1993-1995  N=2,392 | 1996-1999  N=3,244 | 2000-2002  N=4,421 |
|  | OR (95% CI)^*^ | | | | | |
| None  Any  **By condition**  RA  SLE  Sjogren’s syndrome  Systemic sclerosis  Dermatomyositis  Multiple SARDs | Reference  0.76 (0.63-0.91)  0.76 (0.63-0.91)  0.66 (0.30-1.44)  0.84 (0.39-1.77)  0.76 (0.23-2.53)  5.16 (0.66-40.04)  0.70 (0.30-1.60) | Reference  0.84 (0.75-0.94)  0.83 (0.74-0.94)  1.30 (0.82-2.04)  1.08 (0.69-1.71)  0.57 (0.30-1.07)  0.24 (0.02-2.25)  0.64 (0.40-1.03) | Reference  0.88 (0.81-0.95)  0.85 (0.78-0.93)  1.09 (0.81-1.47)  1.06 (0.79-1.42)  1.83 (0.94-3.56)  0.76 (0.28-2.10)  0.75 (0.53-1.06) | Reference  0.95 (0.71-1.27)  0.98 (0.72-1.34)  0.34 (0.04-2.54)  0.81 (0.19-3.46)  1.01 (0.13-7.77)  7.95 (0.44-144.3)  0.86 (0.20-3.74) | Reference  0.88 (0.71-1.08)  0.87 (0.69-1.09)  0.78 (0.28-2.19)  1.42 (0.67-3.02)  0.79 (0.27-2.28)  -------  0.54 (0.35-1.95) | Reference  0.91 (0.78-1.06)  0.93 (0.79-1.09)  0.42 (0.17-1.00)  0.87 (0.46-1.62)  1.03 (0.23-4.58)  0.88 (0.11-6.85)  1.23 (0.73-2.23) |

Note: Blank cell has no subjects

# *Odds ratios (OR) and 95% confidence intervals (CI) are adjusted for age, year of selection, race, mammography, region of residence, income

# and immunosuppressive therapy.
